# Asperpyrone A attenuates RANKL‐induced osteoclast formation through inhibiting NFATc1, Ca^2+^ signalling and oxidative stress

**DOI:** 10.1111/jcmm.14700

**Published:** 2019-10-15

**Authors:** Xi Chen, Chao Wang, Heng Qiu, Yu Yuan, Kai Chen, Zhen Cao, Ren Xiang Tan, Jennifer Tickner, Jiake Xu, Jun Zou

**Affiliations:** ^1^ School of Sports Science Wenzhou Medical University Wenzhou China; ^2^ School of Kinesiology Shanghai University of Sport Shanghai China; ^3^ School of Biomedical Sciences University of Western Australia Perth Western Australia Australia; ^4^ School of Physical Education and Sports Science South China Normal University Guangzhou China; ^5^ State Key Laboratory of Pharmaceutical Biotechnology Institute of Functional Biomolecules Nanjing University Nanjing China

**Keywords:** Asperpyrone A, Ca^2+^ signalling, osteoclast, osteoporosis, reactive oxygen species

## Abstract

Imbalance of osteoblast and osteoclast in adult leads to a variety of bone‐related diseases, including osteoporosis. Thus, suppressing the activity of osteoclastic bone resorption becomes the main therapeutic strategy for osteoporosis. Asperpyrone A is a natural compound isolated from Aspergillus niger with various biological activities of antitumour, antimicrobial and antioxidant. The present study was designed to investigate the effects of Asperpyrone A on osteoclastogenesis and to explore its underlining mechanism. We found that Asperpyrone A inhibited RANKL‐induced osteoclastogenesis in a dose‐dependent manner when the concentration reached 1 µm, and with no cytotoxicity until the concentration reached to 10 µm. In addition, Asperpyrone A down‐regulated the mRNA and protein expression of NFATc1, c‐fos and V‐ATPase‐d2, as well as the mRNA expression of TRAcP and Ctsk. Furthermore, Asperpyrone A strongly attenuated the RNAKL‐induced intracellular Ca^2+^ oscillations and ROS (reactive oxygen species) production in the process of osteoclastogenesis and suppressed the activation of MAPK and NF‐κB signalling pathways. Collectively, Asperpyrone A attenuates RANKL‐induced osteoclast formation via suppressing NFATc1, Ca^2+^ signalling and oxidative stress, as well as MAPK and NF‐κB signalling pathways, indicating that this compound may become a potential candidate drug for the prevention or treatment of osteoporosis.

## 1 INTRODUCTION

In the adults, bone undergoes constant remodelling through the balance of osteoblast‐mediated bone formation and osteoclast‐induced bone resorption.^1^, ^2^ Disruption of the balance may result in various bone‐related diseases, in particular osteoporosis, which is characterized by reduction of bone mass or bone mineral density (BMD), as well as deterioration of the bone structure, leading to fragility fracture.^3^, ^4^ Osteoporosis has become a major social health problem as ageing population increased rapidly all over the world despite currently available treatments.^5^ As derived from the monocyte/macrophage haematopoietic lineage, osteoclast plays an essential role in bone resorption. Thus, suppressing or inhibiting the osteoclast formation or activity was concerned to be the therapeutic potential for preventing the development of osteoporosis. Although bisphosphonates (eg alendronate) are widely prescribed for senile osteoporosis through inhibiting osteoclast formation and activities, the adverse effects limited the use of this drug.^6^ Therefore, screening and finding new natural compounds which had the effects on inhibiting osteoclastogenesis may serve as potential therapies to treat senile osteoporosis and have become an attractive research topics.

Two key cytokines involved in the process of osteoclastogenesis: (a) RANKL (receptor activator of NF‐κB ligand) is an essential cytokine in the process of the osteoclast differentiation, which interacts with RANK to regulate signalling pathways such as NF‐κB, MAPKs and calcium signalling in osteoclasts and then consequently stimulates the downstream factor‐related osteoclastogenesis such as NFATc1 (nuclear factor of activated T cells, cytoplasmic 1) and c‐Fos (a protein of AP‐1 transcription factor required).^7^ (b) M‐CSF (macrophage colony‐stimulating factor) is another crucial cytokine involved in the proliferation and differentiation of monocytes/macrophages, especially in the regulation of the survival and proliferation of pre‐osteoclasts and mature osteoclasts.^8^ Additionally, M‐CSF also up‐regulates RANK levels in BMMs (bone marrow macrophage cells) and then enhances the effects of RANKL stimulation on osteoclast differentiation.^9^

The natural compound of Asperpyrone A was isolated from Aspergillus niger. As one of BNPs (Bis‐naphtho‐γ‐pyrones), it has biological activities including antitumour, antimicrobial and antioxidant.^10^, ^11^ Based on our initial compound screening, we found that Asperpyrone A was a candidate drug for attenuating osteoclast formation. Therefore, we investigated whether Asperpyrone A could inhibit osteoclast formation or activation and explored its effects on RANKL‐induced signalling pathways such as MAPK, NF‐κB, Ca^2+^ signalling and ROS products in the present study. This study also provides evidence that Asperpyrone A may become a potential candidate drug for the prevention or treatment of osteoporosis.

## 2 MATERIALS AND METHODS

### 2.1 Materials

Asperpyrone A, with a purity >99%, was obtained from Professor RenXiang Tan in Nanjing University's (China). DMSO (dimethylsulphoxide), Trizol reagent, FBS (foetal bovine serum), a‐MEM (alpha‐modified minimal essential medium) and Ca^2+^ oscillations assay were purchased from Thermo Fisher Scientific. Recombinant M‐CSF was produced from R&D Systems, and RANKL protein (GST‐rRNAKL) was expressed and purified as previously described.^12^ The MTS cytotoxicity assay and luciferase report assay were purchased from Promega. The antibodies of phosphorylated ERK (p‐ERK), NFATc1, V‐ATPase‐d2, c‐fos, IκB‐α and JNK were obtained from Santa Cruz and the antibodies of P38, phosphorylated P‐38 (P‐P38), phosphorylated JNK(p‐JNK), c‐fos and ERK from Cell Signaling Technology.

### 2.2 Cell culture

Six‐week‐old C57BL/6J mice were used for bone marrow macrophage cells (BMMs) isolation. After flushing the femur and tibia by α‐MEM medium, the cells were cultured in the complete culture medium (10% FBS and 100 U/mL penicillin, 100 g/mL streptomycin and 2 mmol/L L‐glutamine) and supplied with M‐CSF (50 ng/mL). The BMMs were passaged when they reached 70%‐80% confluence, and the 3rd passage was used for further research. RAW264.7 cells were purchased from the American Type Culture Collection and cultured in complete culture medium without M‐CSF. Then, the RAW264.7 cells were prepared for the luciferase reporter assay by stably constructed of the NF‐kB‐ or the NFATc1‐responsive luciferase reporter.^13^

### 2.3 Osteoclastogenesis assay and compounds screening

Bone marrow macrophage cells were seeded into 96‐well plates at a density of 6000 cells/well and cultured with complete medium supplemented with M‐CSF (50 ng/mL) and RANKL (50 ng/mL) for 5 days. TRAcP (tartrate‐resistant acid phosphatase) staining was used for osteoclastogenesis assay. Natural compound screening was performed to detect whether they can inhibit RANKL‐induced osteoclastogenesis using BMM culture (the compounds varying concentrations from 0.5 μmol/L to 10 μmol/L).

### 2.4 Cytotoxicity assays

MTS was used to detect the cell cytotoxicity of candidate compound Asperpyrone A. BMMs were seeded into a 96‐well plate at a density of 6000 cells/well. After overnight, the cells were treated with Asperpyrone A varying concentrations from 0.5 to 10 μmol/L and cultured for 48 hours. Then, MTS solution (20 mL/well) was added and incubated for 2 hours. The 490 nm absorbance was read for every well (Thermo Labsystem Multiscan Spectrum, Thermo Lab System).

### 2.5 Bone resorption assay

Bone resorption assay was used to evaluate the effect of Asperpyrone A on inhibiting osteoclast activity. BMMs were seeded into a 6‐well plate with collagen‐coated at a density of 1 × 10^5^ cells/well and induced osteoclastogenesis by M‐CSF and RANKL. When the mature osteoclasts were generated, cells were detached and seeded into 96‐well plates with hydroxyapatite‐coated (Corning, Inc) with the same number of cell per well; then, cells were cultured in complete medium with M‐CSF and RANKL for 48 hours. Half of the wells were used to assess the resorbed areas by bleaching the cells; the remaining half of the wells were used to perform TRAcP staining to assess the number of mature osteoclasts. ImageJ software for Windows (version 1.49) was used to evaluate the resorbed area of hydroxyapatite surface according to the photographs captured by microscopy.

### 2.6 Osteoblast cell culture and differentiation assays

The osteoblast cells were obtained from the calvaria of neonatal mice as previously described.^14^ In briefly, collagenase (0.1%) and dispase (0.2%) were used to digest the calvaria tissue, and then, the isolated cells were cultured in DMEM with 10% FBS, 100 U/mL penicillin and 100 g/mL streptomycin. The 3rd passage was used for osteoblast differentiation assay. Osteoblast cells were seeded in 48‐well plate and cultured with 50 µg/mL ascorbic acid, 10 mmol/L β‐glycerophosphate and 10^−7^ mmol/L dexamethasone (DXM). The cells were treated with BMP, 2.5 and 5 μmol/L Asperpyrone A, respectively. ALP staining was performed for bone differentiation assay on day 7.

### 2.7 Luciferase reporter assays

The RAW264.7 cells were used for luciferase reporter assays as the previous study.^13^ In brief, the RAW264.7 cells with either the NF‐kB‐ or the NFATc1‐responsive luciferase reporter were prepared and seeded into 48‐well plates, respectively. RANKL stimulation (50 ng/mL) followed 1 hour after pre‐treated with various concentrations of Asperpyrone A. Luciferase activity was detected according to the protocol of luciferase reporter assays (Promega).

### 2.8 Reverse transcription (RT)‐PCR analysis

The BMMs were stimulated with M‐CSF (50 ng/mL) and RANKL (50 ng/mL) in the presence or not of Asperpyrone A for 1, 3 and 5 days, respectively; then, Trizol was used to isolate the total RNA of cells. The cDNA was synthesized from the RNA template, and the quantitative real‐time PCR reactions were performed. The Ct value (cycle threshold) was used to assess the gene expression according to the 2^−ΔΔt^ method.^15^ The expression of β‐actin and B2M mRNA was used for normalization. The detailed information of the primers used in RT‐PCR analysis was shown in Table 1.

**Table 1:** The primer sequences for real‐time PCR

Genes	Forward (5'‐3')	Reverse (3'‐5')
NFATc1	CAACGCCCTGACCACCGATAG	GGCTGCCTTCCGTCTCATAGT
c‐Fos	GCGAGCAACTGAGAAGAC	GCGAGCAACTGAGAAGAC
V‐ATPase‐d2	GTGAGACCTTGGAAGACCTGAA	GAGAAATGTGCTCAGGGGCT
TRAcP	TGTGGCCATCTTTATGCT	GTCATTTCTTTGGGGCTT
Ctsk	GGGAGAAAAACCTGAAGC	ATTCTGGGGACTCAGAGC
β‐actin	CACTGTGCCCATCTACGA	TGATGTCACGCACGATTT
B2m	TTCTGGTGCTTGTCTCACTGA	CAGTATGTTCGGCTTCCCATTC

### 2.9 Western blotting

The BMMs were cultured in 6‐well plates with M‐CSF (50 ng/mL) until the cells reached confluence. The cells were pre‐treated with Asperpyrone A or not and then stimulated with RANKL (50 ng/mL) for 10, 20,30 and 60 minutes, respectively. The protein was harvested by RIPA lysis buffer, and the concentration of each sample was equalled (BCA Protein Assay Kit). Each sample's protein was separated by SDS‐PAGE (sodium dodecyl sulphate‐polyacrylamide gel electrophoresis) and transferred to PVDF membranes. The membranes were blocked with 5% fat‐free milk for 1 hour and incubated with primary antibodies at 4°C overnight. After removal of the primary antibodies, the membranes were subsequently incubated with the secondary antibody with HRP (horse‐radish peroxidase)‐conjugated for 2 hours at room temperature. The immunoreactive signals were detected by ECL reagent kit on an Image‐quant LAS 4000 (GE Healthcare). The grey value was calculated by the software of Image J for Windows (version 1.49).

### 2.10 Intracellular Ca^2+^ oscillation assay

Ca^2+^ oscillations were investigated by Fluo4‐AM Kit (Thermo Fisher Scientific) as previously reported.^16^ In brief, the BMMs were seeded into a 48‐well plate, and 2.5 or 5 μmol/L of Asperpyrone A was added when the cells were stimulated with M‐CSF and RANKL for 24 hours. After washed by an assay buffer, the cells were incubated in Fluo4 staining solution at 37°Cfor 45 minutes. Then, changed assay buffer incubated for 20 minutes at room temperature. Visualization of fluorescence was detected by the fluorescent microscope (Nikon) for 3 minutes (every 2 seconds captured one image) at an excitation wavelength of 488 nm. Ca^2+^ oscillation was calculated by the difference between the peak and baseline fluorescence intensity in the cells which had more than two peaks of fluorescence intensity.

### 2.11 Intracellular ROS production detection

As described previously,^17^ the BMMs were pre‐treated with Asperpyrone A (2.5 or 5 µmol/L) or not, followed by M‐CSF and RANKL (50 ng/mL), and then incubated in H2DCFDA for 1 hour. As DCF (2',7'‐dichlorofluorescein) converted from H2DCFDA when oxidation showed highly fluorescent, confocal microscope (NIKON A1Si) was used to detect the fluorescence of DCF in the cells and the mean fluorescence intensity (ROI) of ROS‐positive cells was analysed using Image J software.

### 2.12 Statistical analysis

All the results are presented as the mean ± standard deviation (SD), One‐way ANOVA test was used for the comparing the means of all the groups, followed by Bonferroni's post hoc test for every two groups analysis. Statistical software IBM SPSS for Windows (version 21) was used to perform all the statistical analyses, and GraphPad Prism 5 (version5.01) was used to prepare the bar figures. *P < *.05 was considered as significant differences.

## 3 RESULTS

### 3.1 Asperpyrone A inhibited RANKL‐induced osteoclast formation

**Figure 1:**
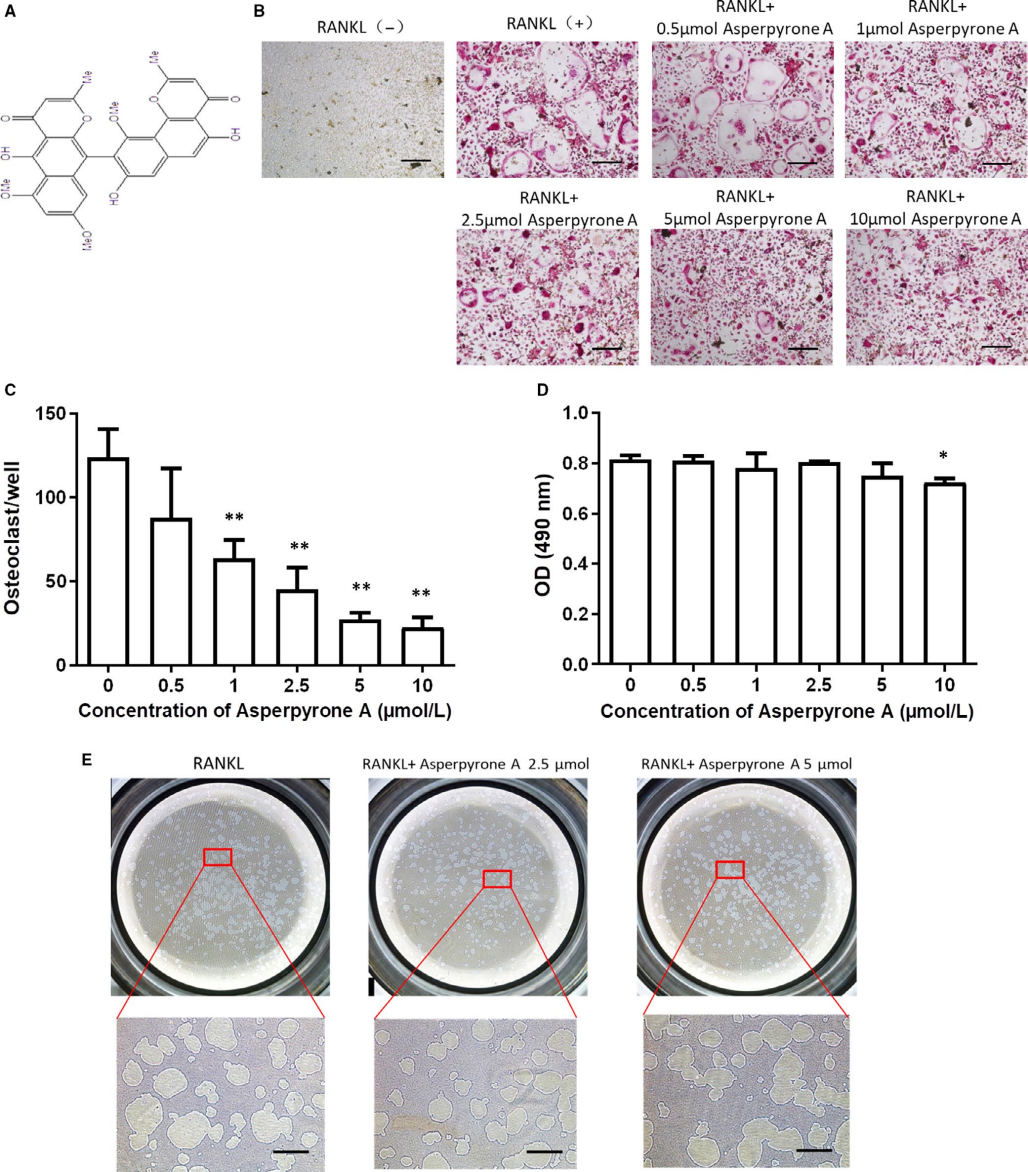
Asperpyrone A inhibits RANKL‐induced osteoclastogenesis. A, The molecular structure of Asperpyrone A. B, The representative images of TRAcP staining after RNAKL‐induced osteoclastogenesis for 5 d with or without Asperpyrone A. C, The quantification of osteoclast number according to TRAcP staining. (n = 3) D, MTS assay after treating with varying concentrations of Asperpyrone A for 48 h. (n = 3) E, The representative images of hydroxyapatite resorption in BMMs when treated with or without Asperpyrone A. All the data are presented as mean ± SD. *P < .05, **P < .01 compared with the positive group (with RNAKL and M‐CSF but without Asperpyrone A treated). Scale bar = 200μm

The results of compounds screening showed that Asperpyrone A (Figure 1A) had a potent inhibitory effect on osteoclast formation. At concentration of 1 μmol/L, Asperpyrone A could suppress RANKL‐induced osteoclast formation, and increasing concentration of Asperpyrone A had more efficacy (1 to 10 μmol/L) in suppressing osteoclastogenesis (Figure 1B,C). Then, the toxicity assay was performed, and Asperpyrone A had shown no cytotoxicity on BMM cells until the concentration reached 10 μmol/L, only with slight cytotoxicity when the concentration reached 10 μmol/L (Figure 1D).

Interestingly, the Asperpyrone A exhibited no significant inhibitory effect on osteoclast activity as the bone resorption assay showed that no more resorption area was found when the concentration of Asperpyrone A reached 5 μmol/L (Figure 1E). Thus, Asperpyrone A mainly affects osteoclast formation.

### 3.2 Asperpyrone A attenuated the expression of the osteoclast‐related genes

**Figure 2:**
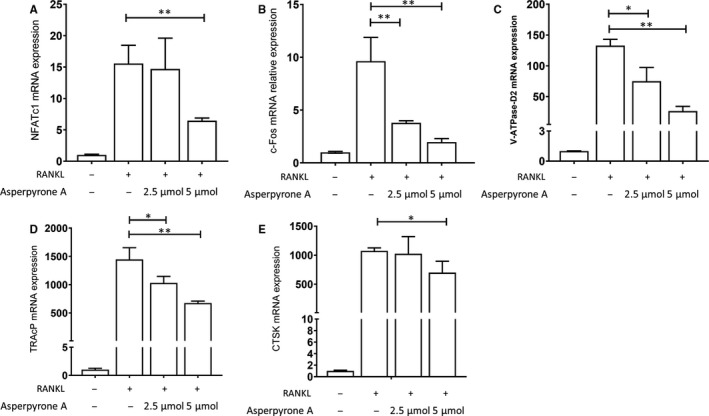
Asperpyrone A inhibits the mRNA expression of osteoclast marker genes. Gene expression was normalized to β‐actin and B2m. A, The relative mRNA expression of NFATc1. B, The relative mRNA expression of c‐fos. C, The relative mRNA expression of V‐ATPase‐d2. D, The relative mRNA expression of TRAcP. E, The relative mRNA expression of Ctsk. All the data are presented as mean ± SD. (n = 3) *P < .05, **P < .01

We then explored the expression of osteoclast‐specific marker genes in BMMs during osteoclast formation with or without the presence of Asperpyrone A. We found that treatment with 5 μmol/L of Asperpyrone A down‐regulated the mRNAs expression of NFATc1, c‐fos, V‐ATPase‐d2 (Vacuolar‐type H+‐ATPase d2), TRAcP and Ctsk in BMMs during osteoclast formation induced by RANKL, and even 2.5 μmol/L of Asperpyrone A inhibited c‐fos, V‐ATPase‐d2 and TRAcP mRNA expression (Figure 2).

### 3.3 Asperpyrone A down‐regulated osteoclast‐related proteins and NFATc1 activity

**Figure 3:**
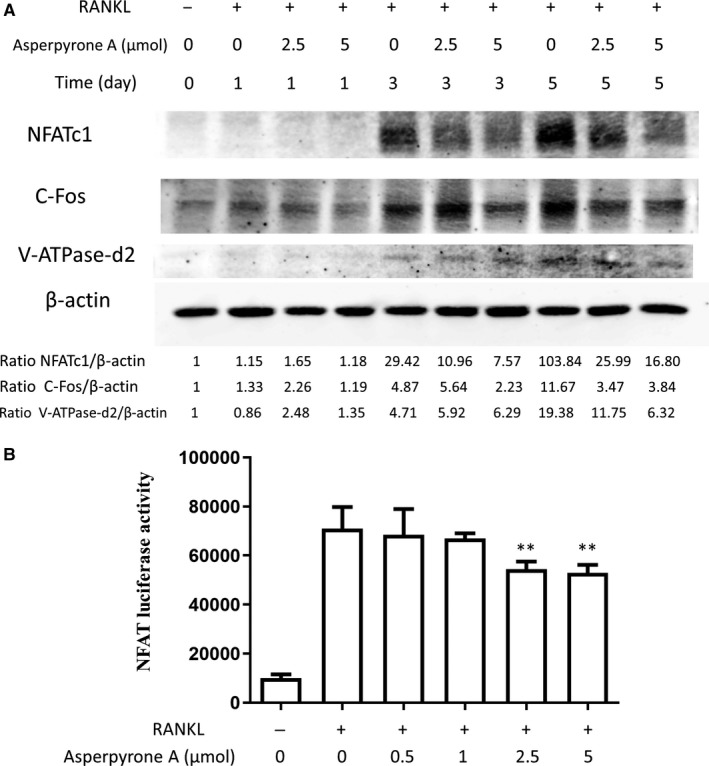
Asperpyrone A down‐regulates osteoclast‐related proteins and NFATc1 activity. A, Western blot was used to detect the protein expression of NFATc1, c‐FOS and V‐ATPase‐d2 in BMMS when pre‐treated with or without 2.5 and 5 μmol/L Asperpyrone A. B, NFAT luciferase activity was investigated when pre‐treated with varying concentration of Asperpyrone A in RAW264.7 cells (n = 3). *P < .05, **P < .01 compared with the positive group (with RNAKL but without Asperpyrone A treated)

The osteoclast‐related proteins including NFATc1, c‐fos and V‐ATPase‐d2 were investigated in BMMs when induced by RANKL for 5 days. The findings demonstrated that the protein level of NFATc1, c‐fos and V‐ATPase‐d2 significantly increased during osteoclastogenesis, whereas Asperpyrone A down‐regulated the protein level of NFATc1, c‐fos and V‐ATPase‐d2 either with the concentration of 2.5 or 5 μmol/L, and 5 μmol/L exhibited more efficacy (Figure 3A).

NFATc1 activity also was evaluated in RAW264.7 cells by luciferase reporter assay. Consistent with the mRNA and protein level in BMMs, the results showed that NFATc1 activity in RAW264.7 cells was also suppressed by Asperpyrone A either with 2.5 or 5 μmol/L concentration (Figure 3B).

### 3.4 Asperpyrone A down‐regulated RANKL‐induced MAPK and NF‐κB signalling pathways

**Figure 4:**
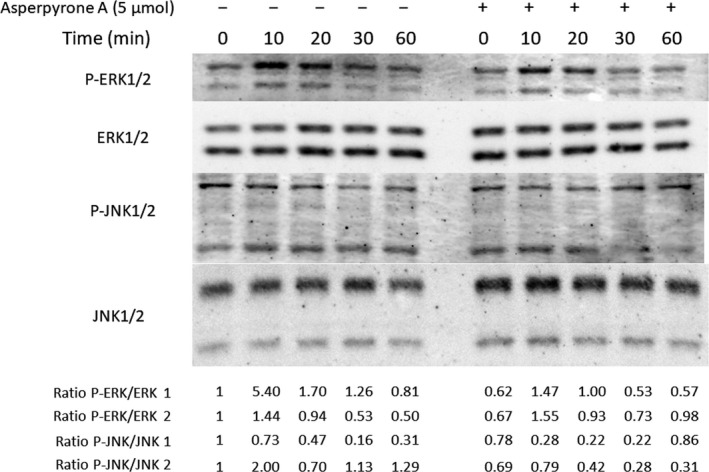
Asperpyrone A down‐regulates ERK and JNK signalling pathways. Western blot was used to detect the protein expression of p‐ERK, ERK, p‐JNK and JNK in BMMs when pre‐treated with or without Asperpyrone A

**Figure 5:**
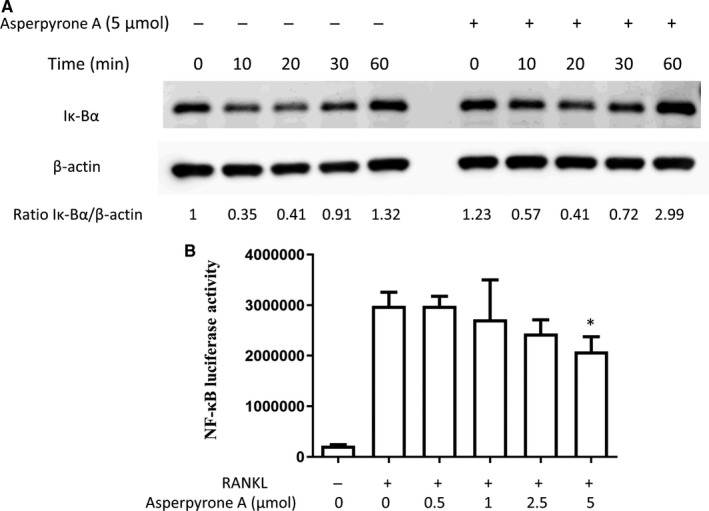
Asperpyrone A suppresses NF‐κB signalling pathway. A, The results of Western blot showed that IκBα protein degradation was attenuated in BMMs when pre‐treated with Asperpyrone A. B, NF‐κB luciferase activity was investigated when pre‐treated with varying concentration of Asperpyrone A in RAW264.7 cells (n = 3). *P < .05, compared with the positive group (with RNAKL but without Asperpyrone A treated)

To further understand the mechanism of Asperpyrone A on suppressing RANKL‐induced osteoclast differentiation in BMMs, MAPK and NF‐κB signalling pathways were explored by Western blot and Luciferase reporter assay. As shown in Figure 4, the ratio of p‐ERK to ERK (p‐ERK/ERK) and p‐JNK to JNK (p‐JNK/JNK) BMMs decreased when treated with 5 μmol/L of Asperpyrone A during RANKL‐induced osteoclast differentiation, which indicated MAPK signalling pathways was suppressed by Asperpyrone A. Moreover, Asperpyrone A also had an inhibitory effect on NF‐κB pathway during osteoclastogenesis as Iκ‐Bα degradation was attenuated when treated with Asperpyrone A in BMMs (Figure 5A). Consistent with the results of Western blot, luciferase reporter assay also exhibited the suppressing effect of Asperpyrone A on NF‐κB activity in RAW264.7 cells.

### 3.5 Asperpyrone A inhibited RANKL‐induced Ca^2+^ oscillations

**Figure 6:**
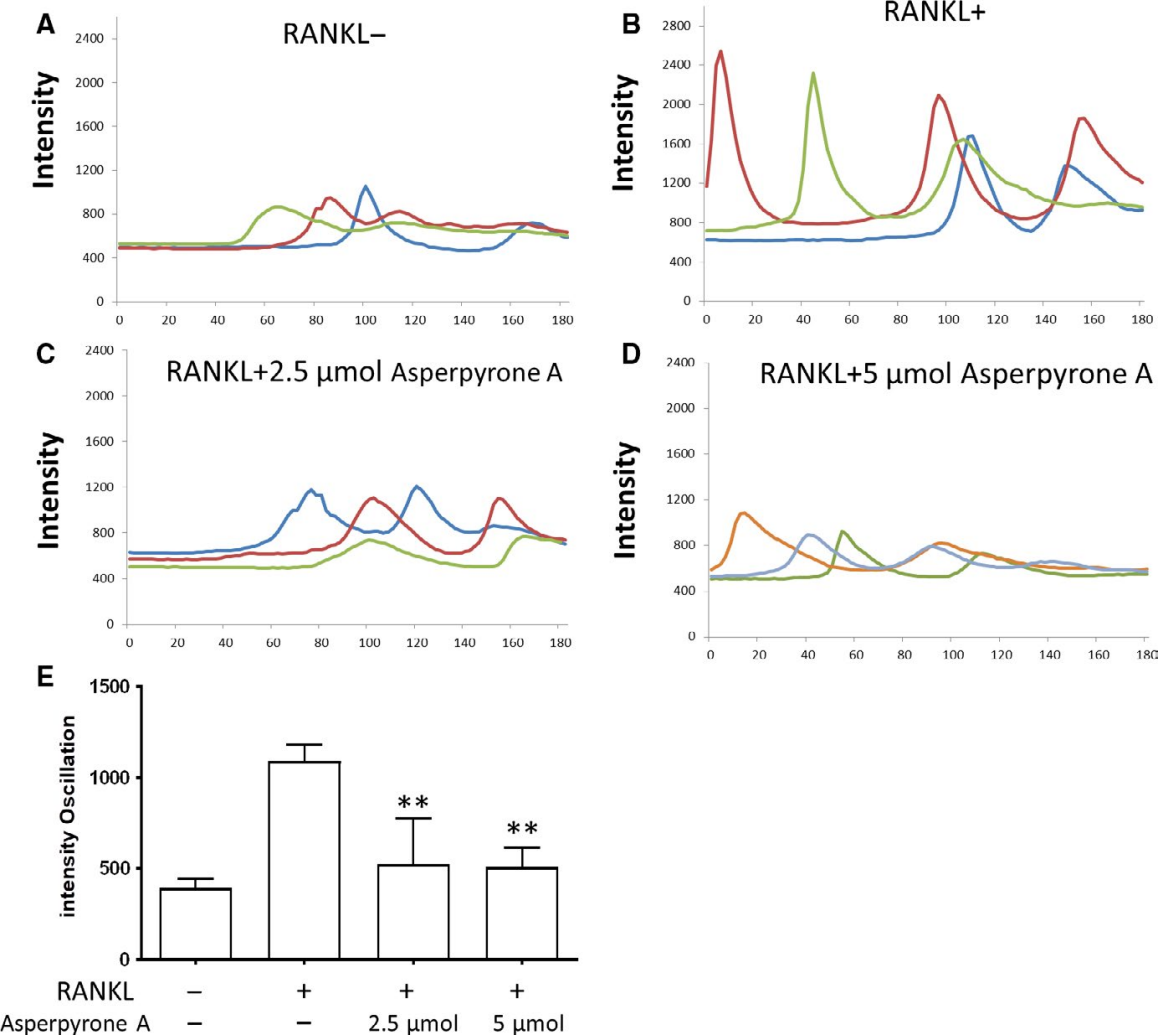
Asperpyrone A attenuates the intensity of RANKL‐induced Ca^2+^ oscillation. (A‐D) The representative image of the intensity of Ca^2+^ fluctuations within 3 minutes after 24 hours pre‐treated with RANKL and Asperpyrone A. E, The quantification of the intensity of Ca^2+^ oscillation per cell (the data showed as mean ± SD. n = 3). **P < .01 compared with the positive group

RANKL stimulates Ca^2+^ oscillations in BMM cells through activation of the Ca^2+^ signal pathways. In the present study, we discovered that the intensity of Ca^2+^ oscillations in BMMs increased and Asperpyrone A had the inhibit effectory during RANKL‐induced osteoclast formation (Figure 6). It appears that both 2.5 and 5 μmol/L concentration of Asperpyrone A exhibited a similar efficacy.

### 3.6 Asperpyrone A suppressed RANKL‐induced intracellular ROS products

**Figure 7:**
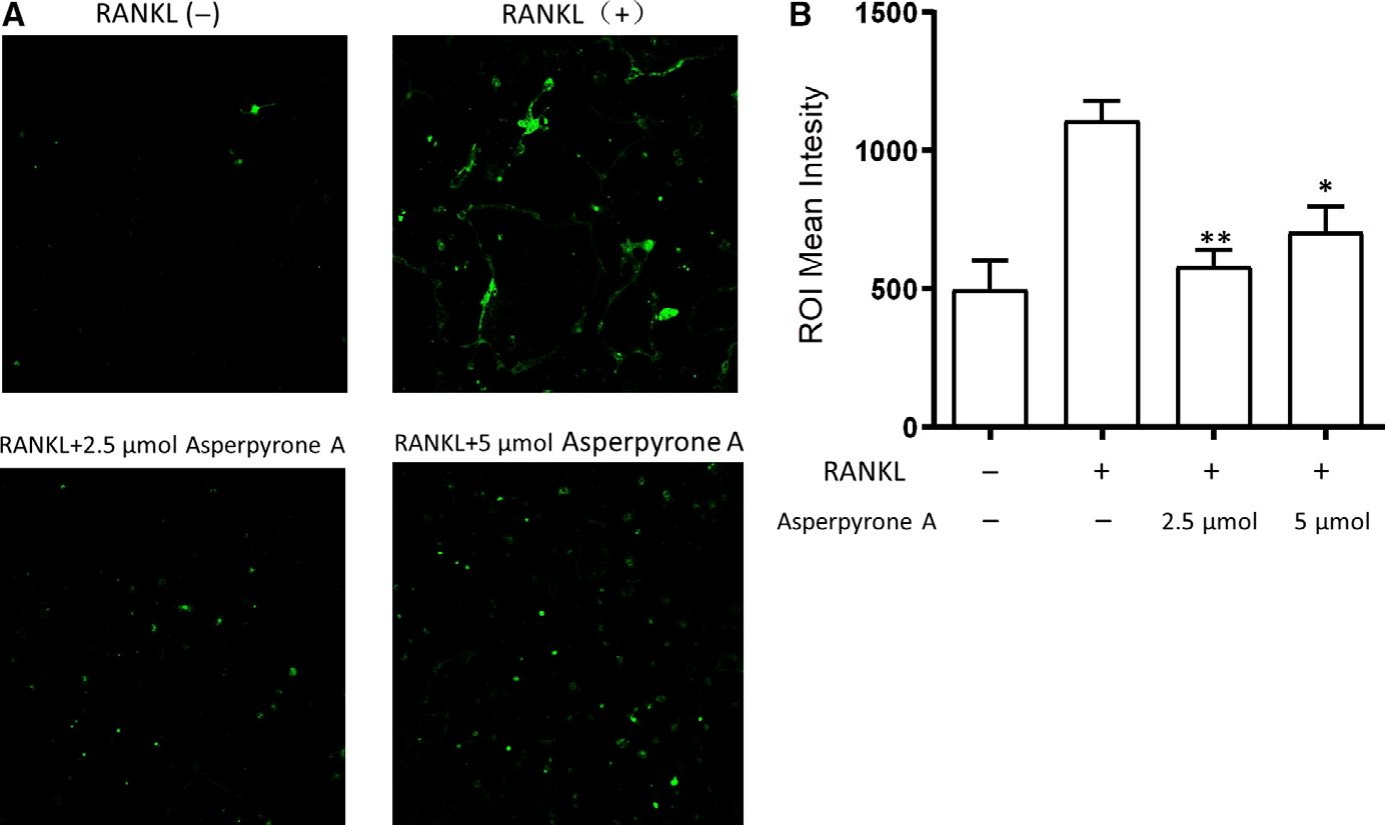
Asperpyrone A suppresses RANKL‐induced intracellular ROS production. A, The representative images of ROS production induced by RANKL in BMMs when pre‐treated with or without Asperpyrone A. B, The quantification of ROS production was calculated. (n = 3). The data are presented as mean ± SD. (n = 3). *P < .05, **P < .01 compared with the positive group (with RNAKL and M‐CSF but without Asperpyrone A treated). Scale bar = 200 µm

As RANKL stimulation increases ROS production in BMMs during osteoclast differentiation,^17^ we then investigated whether Asperpyrone A could reduce ROS products in BMMs during RANKL‐induced osteoclast formation. In this study, the results of fluorescent signal which reflects the ROS products showed increased during RANKL‐induced osteoclast formation and reduced when treated with either 2.5 or 5 μmol/L concentration of Asperpyrone A (Figure 7), which indicated that Asperpyrone A increased the ability to clear intracellular ROS products during RNAKL‐induced osteoclastogenesis.

### 3.7 Asperpyrone A had no effect on the osteoblast differentiation

The result of ALP staining showed that there was no significant difference between Asperpyrone A (2.5 and 5 μmol/L) and control group (Figure S1), indicating that Asperpyrone A did not affect the osteoblast differentiation.

## 4 DISCUSSION

As RANKL has been identified as one of the critical cytokines that regulates osteoclast formation and activity,^18^, ^19^ inhibiting RANKL‐induced osteoclastogenesis was considered to be a potential therapeutic strategy for osteoporosis. For example, Zhou et al demonstrated that dihydroartemisinin suppressed both osteoclast formation and resorption in vitro, as well as reversed the bone loss in ovariectomized mice.^13^ Song et al showed that eriodictyol was potentially useful for the prevention of osteoporosis through inhibiting osteoclast formation and function.^16^ Achyranthes bidentata polysaccharide, berberine sulphate, nitidine chloride, artesunate and so on were also found to be potential therapeutic candidates for the prevention or treatment of osteoporosis.^20^, ^21^, ^22^, ^23^ Other studies also investigated the osteoporotic effects of compounds such as magnolol and polysaccharides.^24^, ^25^, ^26^, ^27^, ^28^

As one of BNPs, the compound of Asperpyrone A was isolated from Aspergillus niger with various biological activities including antitumour, antimicrobial and antioxidant.^10^, ^11^ Its effects on attenuating RANKL‐induced osteoclast formation were identified by our compound screening assay using TRAcP staining. Therefore, we further investigated the effects of Asperpyrone A on suppressing RANKL‐induced osteoclast formation and its cellular mechanisms in the present study. The findings demonstrated that Asperpyrone A significantly decreased both the number and the size of osteoclast, but had no inhibitory effect on the osteoclast function, which indicated Asperpyrone A mainly affects osteoclast formation but not osteoclast activity. In addition, the results of MTS assay exhibited no cytotoxicity on BMM cells until the concentration reached 10 μmol/L. Furthermore, the result of ALP staining showed that the Asperpyrone A had no effect on osteoblast differentiation. These findings suggest that Asperpyrone A has a major effect on osteoclasts and could be a potential candidate anti‐resorptive drug for osteoporosis.

Then, we explored the mechanisms by which Asperpyrone A inhibited osteoclast formation. NFATc1 and c‐fos were identified as the critical regulator in the process of osteoclast formation.^29^ In addition, NFATc1 can amplify its effectiveness via improving other osteoclastogenesis‐related transcription factors in an auto‐amplification loop, including c‐fos, NF‐κB and NFATc2.^30^ Thus, we then investigated the effects of Asperpyrone A on NFATc1 and c‐fos, and found that Asperpyrone A attenuated the expression of the mRNA and protein of NFATc1 and c‐fos in BMMs induced by RANKL. The analysis of luciferase report gene assay also indicates that Asperpyrone A inhibited NFAT activation induced by RANKL in RAW264.7 cells. Moreover, previous studies also showed that NFATc1 can stimulate osteoclasts to express specific proteins which play critical roles in bone matrix resorption, including V‐ATPase‐d2, Ctsk (Cathepsin K) and TRAcP.^31^, ^32^ Therefore, we also investigated the mRNA expression of TRAcP, V‐ATPase‐d2 and Ctsk, and the results showed that the expression of the above genes was also suppressed when treated with Asperpyrone A during RANKL‐induced osteoclast formation. These results exhibited that Asperpyrone A was a potential therapeutic candidate for the prevention of osteoporosis through inhibiting the expression of NFATc1 and other related transcription factors during osteoclastogenesis.

MAPK signalling pathways including ERK, JNK, P38 and NF‐κB signalling pathway were known as the important signal transduction pathways in the process of osteoclast formation.^33^, ^34^ It was also shown that the signalling pathways of ERK, JNK and P38 activated the expression of NFATc1 and c‐fos in osteoclast,^7^, ^32^, ^35^ and then triggered the downstream osteoclastogenesis‐related gene such as Ctsk, V‐ATPase‐d2 and TRAcP.^36^, ^37^ In this study, the results demonstrated that Asperpyrone A attenuated the activation of ERK and JNK signalling pathways during RANKL‐induced osteoclast formation. In addition, the findings also exhibited that Asperpyrone A inhibited the NF‐κB signalling cascade during the process of osteoclastogenesis as the level of IκBα protein degradation decreased and NF‐κB activity measured by the luciferase report gene assay in RAW264.7 cells decreased when pre‐treated with Asperpyrone A. Taken together, Asperpyrone A suppressed the expression of NFATc1, c‐fos, Ctsk, V‐ATPase‐d2 and TRAcP in osteoclasts via the signalling pathways of MAPK/NF‐κB/NFATc1/c‐fos. Furthermore, Ca^2+^ signalling can induce the activation of NFATc1, and long‐lasting Ca^2+^ oscillation was observed during the process of osteoclastogenesis.^38^, ^39^ Therefore, intracellular Ca^2+^ oscillation is crucial for osteoclast formation. In this study, we observed that Asperpyrone A significantly reduced the intensity of Ca^2+^ oscillation both in the concentration of 2.5 and 5 μmol/L, suggesting that down‐regulation of Ca^2+^ signalling by Asperpyrone might also contribute to the inhibitory effects of NFATc1 and subsequently to RANKL‐induced osteoclast formation.

Recent studies have elucidated that intracellular ROS (reactive oxygen species) is involved in the processing of osteoclast formation, and high level of ROS was found in osteoclast precursors when stimulated by RANKL.^17^, ^40^, ^41^ Other studies demonstrated that osteoporotic postmenopausal women had high oxidative stress index when compared to the healthy group,^42^ suggesting a high level of oxidative stress might contribute to the development of osteoporosis and suppressing oxidative stress would be a potential treatment for osteoporosis. Therefore, we investigated the ROS level in the process of RANKL‐induced osteoclast formation and discovered that Asperpyrone A suppressed the ROS production in a dose‐dependent manner. Previous studies had shown that Asperpyrone A hugely inhibited COX‐2 activities and exhibited an antioxidant effect,^43^ which might contribute to the inhibition of the ROS level. Moreover, ROS productions can also stimulate the MAPK and NF‐κB activation,^44^, ^45^, ^46^ indicating Asperpyrone A suppressed MAPK and NF‐κB activation in the present study might through attenuating the intracellular ROS production during RANKL‐induced osteoclast formation.

Previous studies showed that natural compounds not only suppressed the osteoclast formation in vitro, but also reversed the bone loss in ovariectomized (OVX) model mice.^47^, ^48^, ^49^ One of the limitations in this study was that the animal experiments had not been performed. However, based on the evidence of Asperpyrone A inhibited osteoclast formation and did not affect osteoblast differentiation, it is highly possible that Asperpyrone A should be effective for the treatment of osteoclast ‐related conditions such as OVX‐induced osteoporosis.

Taken together, our study had shown that natural compound Asperpyrone A inhibited RNAKL‐induced osteoclast formation via suppressing the intracellular Ca^2+^ signalling and ROS level, subsequently leading to attenuating MAPK and NF‐κB signalling pathways as well as the exepression of NFATc1 and other osteoclast‐related factors. In addition, Asperpyrone A had little effect on osteoblast differentiation. These findings might provide evidence for the use of Asperpyrone A as a novel candidate anti‐resorptive drug for the therapeutic treatment or prevention of osteoporosis.

## CONFLICTS OF INTEREST

The authors declare no conflict of interest.

## AUTHOR CONTRIBUTIONS

Chen X and Wang C performed experiments and wrote the manuscript; Qiu H and Yuan Y performed experiments; Chen K and Cao Z analysed the data and prepared the Figures; Tickner J, Xu J and Zou J designed and guided all the experiments. All authors reviewed the manuscript. Tickner J, Xu J and Zou J revised the manuscript.

## Data Availability

The data used to support the findings of this study are available from the corresponding author upon request.

## References

[1] Rodan GA . Bone homeostasis. Proc Natl Acad Sci USA. 1998;95:13361‐13362. 9811806 10.1073/pnas.95.23.13361 PMC33917

[2] Takayanagi H . Interaction between the immune system and bone metabolism: an emerging field of osteoimmunology. Proc Jpn Acad Ser B Phys Biol Sci. 2007;83:136‐143. 10.2183/pjab.83.136 PMC3756876 24019592

[3] NIH Consensus Development Panel on Osteoporosis Prevention Diagnosis, and Therapy . Osteoporosis prevention, diagnosis, and therapy. JAMA. 2001;285:785‐795.11176917

[4] Bliuc D , Alarkawi D , Nguyen TV , et al. Risk of subsequent fractures and mortality in elderly women and men with fragility fractures with and without osteoporotic bone density: the Dubbo Osteoporosis Epidemiology Study. J Bone Miner Res. 2015;30(4):637‐646. 25359586 10.1002/jbmr.2393

[5] Wade SW , Strader C , Fitzpatrick LA , et al. Estimating prevalence of osteoporosis: examples from industrialized countries. Arch Osteoporos. 2014;9:182. 24847682 10.1007/s11657-014-0182-3

[6] Kennel KA , Drake MT . Adverse effects of bisphosphonates: implications for osteoporosis management. Mayo Clin Proc. 2009;84:632‐637. 19567717 10.1016/S0025-6196(11)60752-0 PMC2704135

[7] Takayanagi H , Kim S , Koga T , et al. Induction and activation of the transcription factor NFATc1 (NFAT2) integrate RANKL signaling in terminal differentiation of osteoclasts. Dev Cell. 2002;3:889‐901. 12479813 10.1016/s1534-5807(02)00369-6

[8] Rosenfeld L , Shirian J , Zur Y , et al. Combinatorial and computational approaches to identify interactions of Macrophage Colony‐stimulating Factor (M‐CSF) and Its Receptor c‐FMS. J Biol Chem. 2015;290:26180‐26193. 26359491 10.1074/jbc.M115.671271 PMC4646268

[9] Takayanagi H . Osteoimmunology: shared mechanisms and crosstalk between the immune and bone systems. Nat Rev Immunol. 2007;7:292‐304. 17380158 10.1038/nri2062

[10] Cai X , Yu Y , Li Q , et al. Asperpyrone F, a new dimeric naphtho‐gamma‐pyrone from the edible fungus *Pleurotus ostreatus* . Nat Prod Res. 2018;33:1953‐1960. 29855204 10.1080/14786419.2018.1481844

[11] Padhi S , Masi M , Panda SK , et al. Antimicrobial secondary metabolites of an endolichenic Aspergillus niger isolated from lichen thallus of Parmotrema ravum. Nat Prod Res. 2019 10.1080/14786419.2018.1544982. 30600725

[12] Xu J , Tan JW , Huang L , et al. Cloning, sequencing, and functional characterization of the rat homologue of receptor activator of NF‐kappaB ligand. J Bone Miner Res. 2000;15:2178‐2186. 11092398 10.1359/jbmr.2000.15.11.2178

[13] Zhou L , Liu Q , Yang M , et al. Dihydroartemisinin, an anti‐malaria drug, suppresses estrogen deficiency‐induced osteoporosis, osteoclast formation, and rankl‐induced signaling pathways. J Bone Miner Res. 2016;31:964‐974. 26684711 10.1002/jbmr.2771

[14] Zhang D , Yi C , Qi S , et al. Effects of carbon nanotubes on the proliferation and differentiation of primary osteoblasts. Methods Mol Biol. 2010;625:41‐53. 20422380 10.1007/978-1-60761-579-8_5

[15] Livak KJ , Schmittgen TD . Analysis of relative gene expression data using real‐time quantitative PCR and the 2(‐Delta Delta C(T)) Method. Methods. 2001;25:402‐408. 11846609 10.1006/meth.2001.1262

[16] Song F , Zhou L , Zhao J , et al. Eriodictyol inhibits RANKL‐induced osteoclast formation and function via inhibition of NFATc1 activity. J Cell Physiol. 2016;231:1983‐1993. 26754483 10.1002/jcp.25304

[17] Lee NK , Choi YG , Baik JY , et al. A crucial role for reactive oxygen species in RANKL‐induced osteoclast differentiation. Blood. 2005;106:852‐859. 15817678 10.1182/blood-2004-09-3662

[18] Lacey DL , Timms E , Tan HL , et al. Osteoprotegerin ligand is a cytokine that regulates osteoclast differentiation and activation. Cell. 1998;93:165‐176. 9568710 10.1016/s0092-8674(00)81569-x

[19] Kong YY , Yoshida H , Sarosi I , et al. OPGL is a key regulator of osteoclastogenesis, lymphocyte development and lymph‐node organogenesis. Nature. 1999;397:315‐323. 9950424 10.1038/16852

[20] Song D , Cao Z , Huang S , et al. Achyranthes bidentata polysaccharide suppresses osteoclastogenesis and bone resorption via inhibiting RANKL signaling. J Cell Biochem. 2018;119:4826‐4835. 29345352 10.1002/jcb.26682

[21] Zhou L , Song F , Liu Q , et al. Berberine sulfate attenuates osteoclast differentiation through RANKL induced NF‐kappaB and NFAT pathways. Int J Mol Sci. 2015;16:27087‐27096. 26580592 10.3390/ijms161125998 PMC4661856

[22] Liu Q , Wang T , Zhou L , et al. Nitidine chloride prevents OVX‐induced bone loss via suppressing NFATc1‐mediated osteoclast differentiation. Sci Rep. 2016;6:36662. 27821837 10.1038/srep36662 PMC5099608

[23] Wei CM , Liu Q , Song FM , et al. Artesunate inhibits RANKL‐induced osteoclastogenesis and bone resorption in vitro and prevents LPS‐induced bone loss in vivo. J Cell Physiol. 2018;233:476‐485. 28294321 10.1002/jcp.25907

[24] Hwang YH , Ha H , Kim R , et al. Anti‐osteoporotic effects of polysaccharides isolated from persimmon leaves via osteoclastogenesis inhibition. Nutrients. 2018;10:901. 10.3390/nu10070901 PMC6073770 30011853

[25] Hwang YH , Kim T , Kim R , Ha H . Magnolol inhibits osteoclast differentiation via suppression of rankl expression. Molecules. 2018;23:1598. 10.3390/molecules23071598 PMC6100296 30004401

[26] Lu SH , Chen TH , Chou TC . Magnolol Inhibits RANKL‐induced osteoclast differentiation of raw 264.7 macrophages through heme oxygenase‐1‐dependent inhibition of NFATc1 expression. J Nat Prod. 2015;78:61‐68. 25574844 10.1021/np500663y

[27] Hwang YH , Kim T , Kim R , Ha H . The natural product 6‐gingerol inhibits inflammation‐associated osteoclast differentiation via reduction of prostaglandin E(2) levels. Int J Mol Sci. 2018;19(7):2068. 10.3390/ijms19072068 PMC6073224 30013004

[28] Chen N , Gao RF , Yuan FL , Zhao MD . Recombinant human endostatin suppresses mouse osteoclast formation by inhibiting the NF‐kappaB and MAPKs signaling pathways. Front Pharmacol. 2016;7:145. 27313530 10.3389/fphar.2016.00145 PMC4887464

[29] Wagner EF , Matsuo K . Signalling in osteoclasts and the role of Fos/AP1 proteins. Ann Rheum Dis. 2003;62(Suppl 2):ii83‐ii85. 14532157 10.1136/ard.62.suppl_2.ii83 PMC1766737

[30] Aliprantis AO , Ueki Y , Sulyanto R , et al. NFATc1 in mice represses osteoprotegerin during osteoclastogenesis and dissociates systemic osteopenia from inflammation in cherubism. J Clin Invest. 2008;118:3775‐3789. 18846253 10.1172/JCI35711 PMC2564610

[31] Gowen M , Lazner F , Dodds R , et al. Cathepsin K knockout mice develop osteopetrosis due to a deficit in matrix degradation but not demineralization. J Bone Miner Res. 1999;14:1654‐1663. 10491212 10.1359/jbmr.1999.14.10.1654

[32] Matsumoto M , Kogawa M , Wada S , et al. Essential role of p38 mitogen‐activated protein kinase in cathepsin K gene expression during osteoclastogenesis through association of NFATc1 and PU.1. J Biol Chem. 2004;279:45969‐45979. 15304486 10.1074/jbc.M408795200

[33] Sharma SM , Bronisz A , Hu R , et al. MITF and PU.1 recruit p38 MAPK and NFATc1 to target genes during osteoclast differentiation. J Biol Chem. 2007;282:15921‐15929. 17403683 10.1074/jbc.M609723200

[34] Miyazaki T , Katagiri H , Kanegae Y , et al. Reciprocal role of ERK and NF‐kappaB pathways in survival and activation of osteoclasts. J Cell Biol. 2000;148:333‐342. 10648566 10.1083/jcb.148.2.333 PMC2174281

[35] Ikeda F , Nishimura R , Matsubara T , et al. Critical roles of c‐Jun signaling in regulation of NFAT family and RANKL‐regulated osteoclast differentiation. J Clin Invest. 2004;114:475‐484. 15314684 10.1172/JCI19657 PMC503767

[36] Balkan W , Martinez AF , Fernandez I , et al. Identification of NFAT binding sites that mediate stimulation of cathepsin K promoter activity by RANK ligand. Gene. 2009;446:90‐98. 19563866 10.1016/j.gene.2009.06.013

[37] Feng H , Cheng T , Steer JH , et al. Myocyte enhancer factor 2 and microphthalmia‐associated transcription factor cooperate with NFATc1 to transactivate the V‐ATPase d2 promoter during RANKL‐induced osteoclastogenesis. J Biol Chem. 2009;284:14667‐14676. 19321441 10.1074/jbc.M901670200 PMC2682914

[38] Negishi‐Koga T , Takayanagi H . Ca2+‐NFATc1 signaling is an essential axis of osteoclast differentiation. Immunol Rev. 2009;231:241‐256. 19754901 10.1111/j.1600-065X.2009.00821.x

[39] Kuroda Y , Hisatsune C , Nakamura T , et al. Osteoblasts induce Ca2+ oscillation‐independent NFATc1 activation during osteoclastogenesis. Proc Natl Acad Sci USA. 2008;105:8643‐8648. 18552177 10.1073/pnas.0800642105 PMC2438406

[40] Kim H , Lee YD , Kim HJ , et al. SOD2 and Sirt3 control osteoclastogenesis by regulating mitochondrial ROS. J Bone Miner Res. 2017;32:397‐406. 27540894 10.1002/jbmr.2974

[41] Yip KH , Zheng MH , Steer JH , et al. Thapsigargin modulates osteoclastogenesis through the regulation of RANKL‐induced signaling pathways and reactive oxygen species production. J Bone Miner Res. 2005;20:1462‐1471. 16007343 10.1359/JBMR.050324

[42] Altindag O , Erel O , Soran N , et al. Total oxidative/anti‐oxidative status and relation to bone mineral density in osteoporosis. Rheumatol Int. 2008;28:317‐321. 17823800 10.1007/s00296-007-0452-0

[43] Fang W , Lin X , Wang J , et al. Asperpyrone‐type bis‐naphtho‐gamma‐pyrones with COX‐2‐inhibitory activities from marine‐derived fungus Aspergillus niger. Molecules. 2016;21:941 10.3390/molecules21070941 PMC6273789 27447606

[44] Bax BE , Alam AS , Banerji B , et al. Stimulation of osteoclastic bone resorption by hydrogen peroxide. Biochem Biophys Res Commun. 1992;183:1153‐1158. 1567393 10.1016/s0006-291x(05)80311-0

[45] Kim HJ , Chang EJ , Kim HM , et al. Antioxidant alpha‐lipoic acid inhibits osteoclast differentiation by reducing nuclear factor‐kappaB DNA binding and prevents in vivo bone resorption induced by receptor activator of nuclear factor‐kappaB ligand and tumor necrosis factor‐alpha. Free Radic Biol Med. 2006;40:1483‐1493. 16632109 10.1016/j.freeradbiomed.2005.10.066

[46] Kim HS , Nam ST , Mun SH , et al. DJ‐1 controls bone homeostasis through the regulation of osteoclast differentiation. Nat Commun. 2017;8:1519. 29142196 10.1038/s41467-017-01527-y PMC5688089

[47] Li C , Yang Z , Li Z , et al. Maslinic acid suppresses osteoclastogenesis and prevents ovariectomy‐induced bone loss by regulating RANKL‐mediated NF‐kappaB and MAPK signaling pathways. J Bone Miner Res. 2011;26:644‐656. 20814972 10.1002/jbmr.242

[48] Wu X , Li Z , Yang Z , et al. Caffeic acid 3,4‐dihydroxy‐phenethyl ester suppresses receptor activator of NF‐kappaB ligand‐induced osteoclastogenesis and prevents ovariectomy‐induced bone loss through inhibition of mitogen‐activated protein kinase/activator protein 1 and Ca2+‐nuclear factor of activated T‐cells cytoplasmic 1 signaling pathways. J Bone Miner Res. 2012;27:1298‐1308. 22337253 10.1002/jbmr.1576

[49] Yuan FL , Xu RS , Jiang DL , et al. Leonurine hydrochloride inhibits osteoclastogenesis and prevents osteoporosis associated with estrogen deficiency by inhibiting the NF‐kappaB and PI3K/Akt signaling pathways. Bone. 2015;75:128‐137. 25708053 10.1016/j.bone.2015.02.017

