# Supplementary figures and images for: Asperpyrone A attenuates RANKL‐induced osteoclast formation through inhibiting NFATc1, Ca2+ signalling and oxidative stress
Source: J Cell Mol Med. 2019 Oct 15;23(12):8269–79. doi: 10.1111/jcmm.14700 (PMC6850946; doi:10.1111/jcmm.14700)

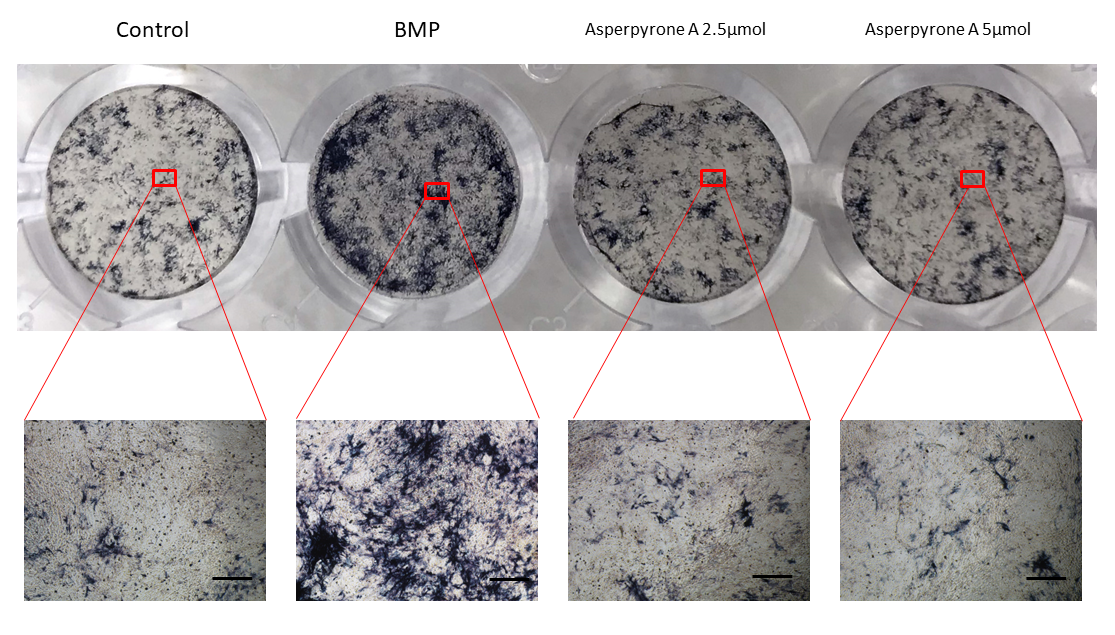

Supplement: Supplementary file 1 [file JCMM-23-8269-s001.tif]
